# Multidimensional assessment of hand hygiene compliance and its association with healthcare-associated infection incidence in intensive care units

**DOI:** 10.1186/s13756-026-01794-9

**Published:** 2026-07-15

**Authors:** Ágnes Csörnyei-Kelemen, Zoltan Csörnyei, Zoltán Balogh

**Affiliations:** 1 Doctoral College of Semmelweis University, 25 Üllői Street, Budapest, 1091 Hungary; 2 National Development Center of Hungary, Budapest, Hungary; 3 Faculty of Health Sciences, Semmelweis University, 17 Vas Street, Budapest, 1428 Hungary

**Keywords:** Hand hygiene, Healthcare-associated infections, Intensive care units, Hand hygiene compliance monitoring, Infection prevention, Compliance variability

## Abstract

**Background:**

Previous studies have reported heterogeneous associations between average hand hygiene (HH) compliance and healthcare-associated infection (HAI) incidence, suggesting that monthly mean compliance alone may not fully characterize clinically relevant patterns of HH performance. We aimed to evaluate whether complementary metrics derived from the same HH dataset could provide additional contextual information on the relationship between HH performance and HAI incidence.

**Methods:**

We conducted a prospective observational study using HH audit data collected across three intensive care units with differing operational and staffing profiles. Monthly HAI incidence data were obtained from a parallel prospective surveillance program conducted by the same infection prevention team. Monthly HH performance was characterized using predefined metrics representing overall compliance level, day-to-day variability, critical low-compliance episodes, and lower-tail performance. These included weighted monthly HH compliance, coefficient of variation, the proportion of critical HH days defined as observed days with daily compliance below 40%, and the 25th percentile of daily compliance values. Associations with HAI incidence were explored using correlation analyses.

**Results:**

Weighted monthly HH compliance demonstrated the strongest pooled inverse association with HAI incidence. Complementary metrics reflecting day-to-day variability, critical low-compliance days, and lower-tail performance were also associated with HAI incidence, although their relative informativeness differed across units. These findings suggest that average compliance remains an important surveillance metric, while additional metrics may help characterize operational instability and episodes of distinctly poor HH performance.

**Conclusions:**

Average HH compliance remains an informative surveillance metric and should not be replaced by complementary indicators. However, measures reflecting day-to-day variability, critical low-compliance days, and lower-tail performance may provide additional operational context for interpreting HH surveillance data. Incorporating multiple HH performance dimensions may support more targeted infection prevention and workforce-management strategies.

## Introduction

Hand hygiene (HH) remains one of the cornerstone interventions for preventing healthcare-associated infections (HAIs) [1] and has been widely promoted through international campaigns and institutional quality improvement programs [2]. Despite strong biological plausibility and extensive implementation efforts, the relationship between measured HH compliance and infection outcomes has proven more complex and heterogeneous than initially anticipated [2–4].

Although numerous studies have demonstrated improvements in HH compliance following targeted interventions, evidence linking higher measured compliance rates directly to reductions in HAI incidence has been inconsistent. In particular, studies relying primarily on aggregated or average HH compliance measures have reported variable, non-significant, or unit-dependent associations. This has raised questions about whether average compliance values, while important, fully characterize the dimensions of HH performance that may be relevant to infection transmission risk [2, 4].

Several explanations have been proposed for the observed heterogeneity in HH–HAI associations [5, 6]. From a methodological perspective, HH compliance is commonly assessed through intermittent direct observation, yielding snapshot measurements that may not fully reflect routine clinical practice. Moreover, summarizing performance using average values may smooth short-term fluctuations and underrepresent episodic failures that occur during periods of increased workload, staffing shortages, or operational strain [7, 8]. Such transient lapses, although brief, may nevertheless have disproportionate consequences for pathogen transmission in high-acuity settings.

The intensive care unit (ICU) is a particularly relevant setting for exploring these issues. ICU care is characterized by high patient acuity, frequent invasive procedures, and complex workflows [9], all of which may amplify the consequences of even brief deviations from infection prevention practices. At the same time, ICU staffing patterns are often dynamic, influenced by workforce shortages, temporary staff deployment, onboarding of newly trained personnel, and staff fatigue or burnout [10]. These factors may contribute to instability in HH performance that may not be fully apparent from monthly average compliance values alone [11–13].

Previous work from the same clinical setting demonstrated that average HH compliance may be associated with HAI incidence in some units but not others, suggesting substantial contextual heterogeneity [14]. Taken together, these observations suggest that inconsistent HH–HAI associations may partly reflect differences in how HH performance is measured, summarized, and interpreted across settings.

Accordingly, there is growing interest in analytical approaches that complement average compliance metrics by incorporating performance variability, lower-tail performance, or threshold-based indicators of critically low compliance. Such approaches do not replace average compliance measures, but may help characterize additional operational dimensions of HH performance. This perspective aligns with systems-based approaches to patient safety, which emphasize that adverse outcomes may arise not only from sustained poor performance but also from brief yet consequential breakdowns in routine processes.

The objective of the present study was therefore to apply multiple predefined and reproducible analytical approaches to a prospectively collected HH dataset and to compare their associations with monthly HAI incidence. By examining three adult ICUs operating under the same institutional leadership but differing in operational and clinical profiles, we aimed to explore whether average and complementary HH metrics together provide a more comprehensive framework for infection prevention surveillance and management.

## Methods

### Study design and setting

We conducted a prospective observational study in a Hungarian university-affiliated teaching hospital. The study was performed in three adult intensive care units (ICUs) operating under the same institutional management structure: one surgical ICU (S.ICU) and two medical ICUs (M.ICU and M.ICU II). Although these units shared identical infection prevention leadership and institutional policies, they differed in operational and staffing characteristics. The study period extended from 1 May 2023 to 30 April 2024.

### Hand hygiene data collection

Hand hygiene (HH) compliance was assessed through direct observation using the World Health Organization (WHO) “Five Moments for Hand Hygiene” framework [15]. Observations were performed on predefined audit days each month by a single trained infection prevention professional using a standardized observation protocol.

To improve representativeness, observations were distributed across different shifts and were planned to include nurses, physicians, and other healthcare workers over time. Sampling was designed to reflect routine clinical practice rather than peak or atypical performance periods. For each observed day, daily HH compliance was calculated as:

$$ \begin{aligned} & {\text{Daily HH compliance}} \\ & = {\text{compliant HH opportunities}} \\ & \quad /{\text{total observed HH opportunities}} \\ \end{aligned} $$

Only audit days directly observed were included in the present analysis. No imputation was performed for non-observed days.

### Monthly hand hygiene performance metrics

Monthly HH performance metrics were calculated separately for each ICU using only observed audit days. Metrics were predefined to capture complementary dimensions of HH performance, including overall compliance level, day-to-day variability, episodes of critically low compliance, and lower-tail performance.

#### Primary metrics

1. Weighted monthly HH compliance. Overall monthly compliance was calculated as:

Weighted monthly HH compliance = total compliant HH opportunities in the month / total observed HH opportunities in the month.

This metric reflects the overall monthly HH compliance level while accounting for differences in the number of observed opportunities across audit days.

2. Performance variability (coefficient of variation, CV). Day-to-day instability in HH performance was expressed as:

CV = SD of daily HH compliance/mean daily HH compliance,

where SD denotes the standard deviation of daily compliance values within a month.

3. Proportion of critical HH days. A critical HH day was defined as an observed day with daily HH compliance < 40%. Critical HH day proportion was calculated as:

Critical HH day proportion = observed days with HH compliance < 40%/observed audit days in the month.

The 40% threshold was selected a priori as a pragmatic operational marker of distinctly poor daily performance. In the present dataset, this threshold approximately corresponded to the lower quartile of observed daily HH compliance values. It was used as an analytical threshold rather than as a universally validated clinical cut-off. Future studies should assess whether alternative thresholds yield comparable findings across different settings.

#### Secondary metric

4. Lower-tail performance (25th percentile, P25). The 25th percentile (P25) of daily HH compliance values within each month was calculated to characterize lower-end daily performance while reducing sensitivity to single extreme observations. P25 was selected as a robust lower-tail descriptor because most ICU-month observations were based on approximately four observed audit days; under these conditions, more extreme lower-tail percentiles are highly sensitive to single daily values and may behave similarly to minimum-like measures.

P25 was classified as a secondary metric because it partly overlaps conceptually with the threshold-based critical HH day proportion, whereas the primary metrics were selected to represent overall compliance level, day-to-day variability, and clinically interpretable low-compliance episodes. Percentiles were calculated using the default linear interpolation method of the statistical software.

### Healthcare-associated infection surveillance

Healthcare-associated infection (HAI) data were obtained from a parallel prospective surveillance program conducted by the same infection prevention team during the same study period. Infections were identified according to standardized Centers for Disease Control and Prevention/National Healthcare Safety Network (CDC/NHSN) surveillance definitions [16].

Monthly HAI incidence rates were calculated as the number of HAIs per 100 patient-days for each ICU. Infection incidence data were temporally aligned with the corresponding monthly HH performance metrics.

### Statistical analysis

The ICU-month was used as the primary analytical unit, yielding 36 ICU-month observations across the three participating units.

Associations between HH performance metrics and monthly HAI incidence rates were explored using Pearson correlation coefficients. 95% confidence intervals were estimated using Fisher’s z transformation.

Because variables were continuous and the study was exploratory, emphasis was placed on effect size, direction, and consistency across alternative metrics rather than on formal hypothesis testing alone. No adjustment was made for multiple comparisons.

Both pooled analyses across all ICUs and exploratory ICU-specific analyses were performed. Pooled analyses included 36 ICU-month observations, whereas ICU-specific exploratory analyses included 12 monthly observations per unit. Pooled analyses were used to improve statistical precision, while unit-specific analyses were undertaken to assess contextual heterogeneity across units with differing operational and staffing profiles. No monthly observations were excluded, and no data were imputed. Statistical analyses were conducted using IBM SPSS Statistics version 20.0 (IBM Corp., Armonk, NY, USA) and Python version 3.12.

### Observer training and observation volume

All HH observations were performed by a single experienced observer with formal training in hand hygiene auditing. The observer was a doctoral candidate (PhD) working within the infection prevention program. The use of a single permanent observer enhanced methodological consistency across units and throughout the study period.

### Ethical considerations

The study was conducted as a non-interventional observational investigation. Ethical approval was granted by the Scientific and Research Ethics Committee of the Hungarian Medical Research Council (ETT TUKEB; approval number: BM/4979-3/2023). The study was performed in accordance with national regulations and institutional policies governing observational research and infection surveillance.

## Results

### Study observations and data structure

Over the 12-month study period, hand hygiene (HH) performance and healthcare-associated infection (HAI) incidence, expressed as HAI episodes per 100 patient-days, were evaluated monthly across three adult intensive care units (ICUs), yielding 36 ICU-month observations. HH observations were completed in all units during each study month, with no missing monthly data.

Across the study period, a total of 7,473 HH opportunities were observed over 154 audit days, comprising 51 audit days in S.ICU, 51 in M.ICU, and 52 in M.ICU II. Most ICU-month observations were based on four audit days, with only modest variation across months.

The three participating units demonstrated distinct HH performance profiles. M.ICU II showed generally higher compliance and lower variability; M.ICU showed persistently lower compliance values; and S.ICU demonstrated intermediate average performance with greater fluctuation over time. These descriptive patterns supported the rationale for examining both pooled and ICU-specific associations, while also indicating that differences in average compliance level and performance stability could not be fully disentangled in unit-specific comparisons.

### Pooled associations between hand hygiene metrics and HAI incidence

**Fig. 1:**
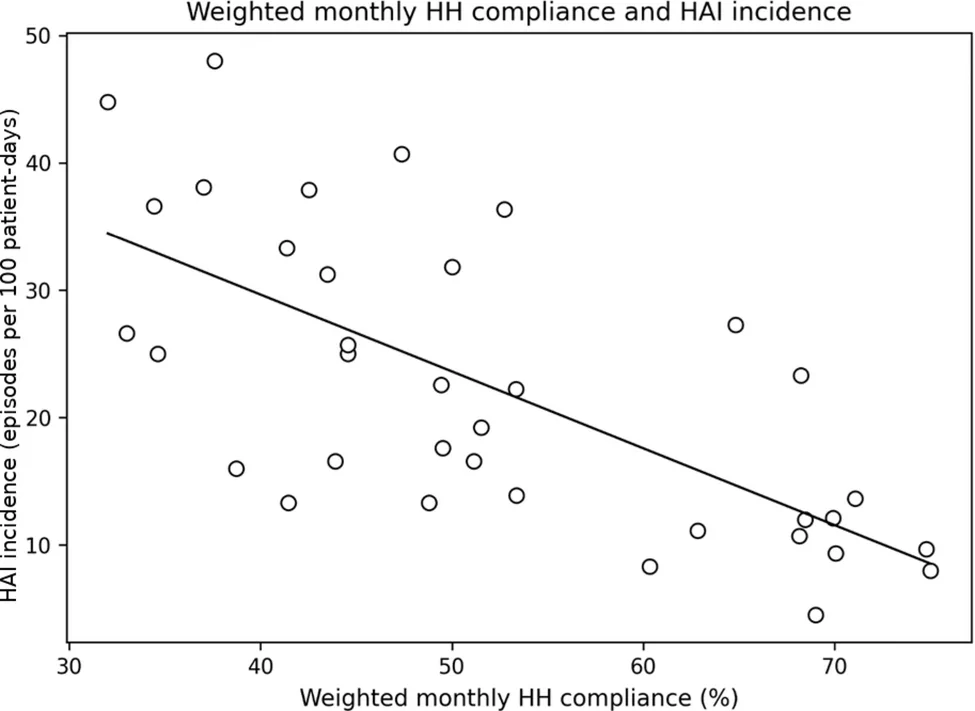
Association between weighted monthly hand hygiene compliance and healthcare-associated infection incidence. Scatter plot showing the relationship between weighted monthly hand hygiene (HH) compliance and monthly healthcare-associated infection (HAI) incidence, expressed as HAI episodes per 100 patient-days, across 36 ICU-month observations. Weighted monthly HH compliance was inversely associated with HAI incidence

In pooled analyses across all 36 ICU-month observations, weighted monthly HH compliance demonstrated the strongest inverse association with monthly HAI incidence (*r* = − 0.685, 95% CI − 0.827 to − 0.460, *p* < 0.001), indicating that higher overall monthly compliance was associated with lower infection rates (Fig. 1).

**Fig. 2:**
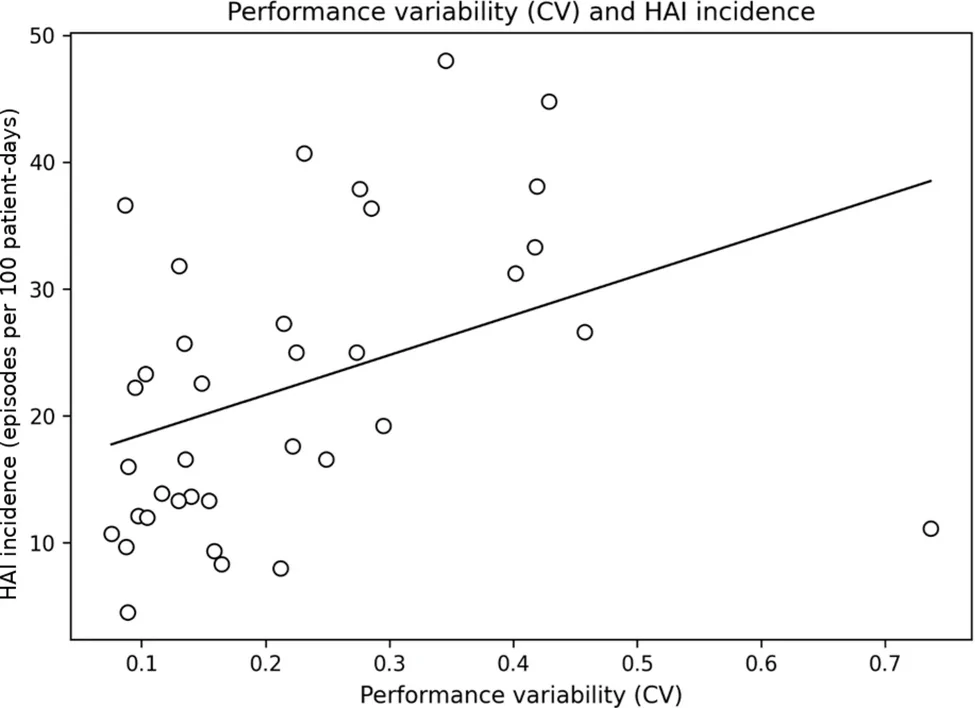
Association between hand hygiene variability and healthcare-associated infection incidence. Scatter plot showing the relationship between day-to-day hand hygiene (HH) variability, expressed as the coefficient of variation (CV), and monthly healthcare-associated infection (HAI) incidence, expressed as HAI episodes per 100 patient-days, across 36 ICU-month observations. Higher HH variability was positively associated with HAI incidence

Day-to-day instability in HH performance, expressed as the coefficient of variation (CV), showed a more moderate positive association with HAI incidence (*r* = 0.389, 95% CI 0.069 to 0.636, *p* = 0.019), suggesting that greater variability in monthly HH performance was associated with higher HAI incidence, although less strongly than weighted monthly HH compliance (Fig. 2).

**Fig. 3:**
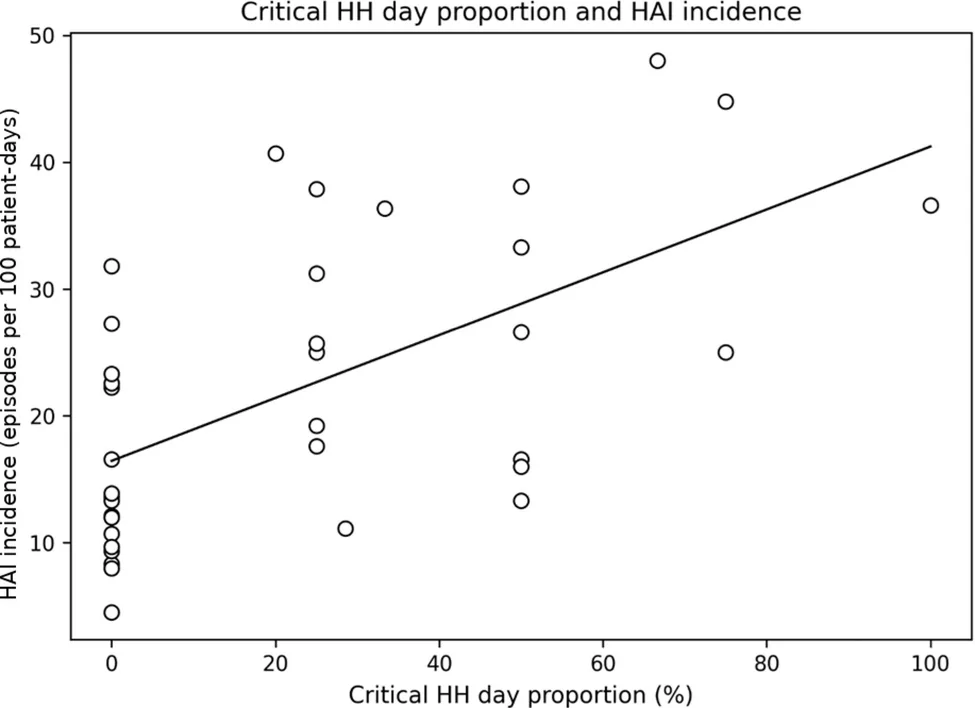
Association between critical hand hygiene days and healthcare-associated infection incidence. Scatter plot showing the relationship between the proportion of critical hand hygiene (HH) days, defined as observed audit days with daily HH compliance below 40%, and monthly healthcare-associated infection (HAI) incidence, expressed as HAI episodes per 100 patient-days, across 36 ICU-month observations. A higher proportion of critical HH days was positively associated with HAI incidence

The proportion of critical HH days, defined as the proportion of observed audit days within a month on which daily HH compliance was below 40%, was positively associated with monthly HAI incidence (*r* = 0.590, 95% CI 0.325 to 0.770, *p* < 0.001). This indicates that months with a greater share of distinctly poor-performance days tended to have higher HAI rates (Fig. 3).

The secondary lower-tail metric, defined as the 25th percentile of daily HH compliance values within each month (P25), also showed a substantial inverse association with HAI incidence (*r* = − 0.615, 95% CI − 0.785 to − 0.360, *p* < 0.001). Thus, months characterized by poorer lower-end daily performance also tended to have higher HAI incidence.

For completeness, the unweighted average of daily compliance values within a month (HH_mean_daily) was also inversely associated with HAI incidence (*r* = − 0.620, 95% CI − 0.788 to − 0.367, *p* < 0.001). This metric differs from weighted monthly HH compliance because it is calculated as the arithmetic mean of observed daily compliance values, whereas weighted monthly HH compliance is calculated as the total number of compliant HH opportunities divided by the total number of observed HH opportunities within the month. Because the weighted measure accounts for differences in observation volume across audit days, it was retained as the principal average-compliance metric. In contrast, the standard deviation of daily compliance values alone (HH_sd) was only weakly associated and not statistically significant (*r* = 0.167, 95% CI − 0.170 to 0.470, *p* = 0.329). Because weighted monthly HH compliance, CV, critical HH day proportion, and P25 were predefined as the main metrics of interest, subsequent interpretation focused on these measures.

**Table 1:** Associations between predefined hand hygiene performance metrics and monthly HAI incidence across 36 ICU-month observations

Hand hygiene metric	Pearson *r*	95% CI for *r*	*p* value
Weighted monthly HH compliance	− 0.685	− 0.827 to − 0.460	< 0.001
HH variability (CV)	0.389	0.069 to 0.636	0.019
Critical HH day proportion (< 40%)	0.590	0.325 to 0.770	< 0.001
Lower-tail performance (P25)	-0.615	− 0.785 to − 0.360	< 0.001

HH, hand hygiene; HAI, healthcare-associated infection; CI, confidence interval; CV, coefficient of variation. HAI incidence was expressed as HAI episodes per 100 patient-days

#### Mean compliance versus complementary metrics

Although weighted monthly HH compliance showed the strongest pooled association with HAI incidence, average compliance alone did not fully characterize the operational differences observed between units or months. ICU-month observations with broadly similar average compliance values could still differ in day-to-day variability, in the proportion of critical low-compliance days, and in lower-tail performance.

The complementary metrics provided different forms of information. The weighted monthly mean represented the overall monthly HH level. The CV captured instability across observed days within the month. The critical HH day proportion identified months with repeated episodes of distinctly poor performance, while P25 characterized the lower end of daily performance more robustly than minimum-value metrics, being less sensitive to isolated outlier days.

Taken together, these results indicate that average compliance and complementary metrics should not be interpreted as mutually exclusive alternatives. Rather, they describe different dimensions of HH performance: average level, day-to-day stability, frequency of critical failures, and lower-end performance.

### ICU-specific exploratory analyses

Because the three ICUs differed in observed HH performance profiles, exploratory ICU-specific analyses were undertaken to assess contextual heterogeneity.

In S.ICU, several metrics showed comparatively strong associations with HAI incidence. Weighted monthly HH compliance was inversely associated with HAI incidence (*r* = − 0.669, 95% CI − 0.898 to − 0.154, *p* = 0.017). HH variability (CV) showed the strongest association in this unit (*r* = 0.746, 95% CI 0.301 to 0.924, *p* = 0.005). The proportion of critical HH days was positively associated with HAI incidence (*r* = 0.618, 95% CI 0.068 to 0.880, *p* = 0.032), and lower-tail performance (P25) also showed an inverse association (*r* = − 0.694, 95% CI − 0.907 to − 0.200, *p* = 0.012). These findings suggest that in S.ICU, several HH performance dimensions, including both average compliance and instability-related metrics, were associated with HAI incidence.

In M.ICU, the same general directions of association were observed, but effect estimates were weaker and confidence intervals wider. Weighted monthly HH compliance was inversely associated with HAI incidence (*r* = − 0.480, 95% CI − 0.826 to 0.130, *p* = 0.114). HH variability (CV) showed a smaller positive association (*r* = 0.370, 95% CI − 0.259 to 0.778, *p* = 0.237). The proportion of critical HH days was positively associated with HAI incidence (*r* = 0.475, 95% CI − 0.136 to 0.824, *p* = 0.119), and P25 also showed an inverse association (*r* = − 0.465, 95% CI − 0.820 to 0.149, *p* = 0.128). Although these ICU-specific associations did not reach conventional significance thresholds, their directions were broadly consistent with the pooled findings.

In M.ICU II, associations were weaker and less consistent. Weighted monthly HH compliance showed a moderate inverse association with HAI incidence (*r* = − 0.465, 95% CI − 0.820 to 0.149, *p* = 0.128), whereas HH variability (CV; *r* = 0.049, 95% CI − 0.540 to 0.606, *p* = 0.879), critical HH day proportion (*r* = 0.105, 95% CI − 0.499 to 0.640, *p* = 0.746), and P25 (*r* = -0.156, 95% CI − 0.670 to 0.459, *p* = 0.629) showed minimal associations with HAI incidence. This pattern is compatible with the descriptively higher baseline HH compliance and lower between-month variability observed in this unit.

These exploratory analyses indicate that the relative informativeness of different HH metrics may vary across units and may depend on local operational context. However, because the three ICUs differed in both average compliance level and stability, these analyses should not be interpreted as evidence that instability-related metrics are independent of, or more important than, average compliance. Rather, they suggest that complementary metrics may help describe additional performance dimensions within specific unit contexts.

**Table 2:** ICU-specific exploratory associations between predefined hand hygiene metrics and monthly HAI incidence

ICU	Metric	Pearson *r*	95% CI for *r*	*p* value
S.ICU	Weighted monthly HH compliance	− 0.669	− 0.898 to − 0.154	0.017
	HH variability (CV)	0.746	0.301 to 0.924	0.005
	Critical HH day proportion (< 40%)	0.618	0.068 to 0.880	0.032
	Lower-tail performance (P25)	− 0.694	− 0.907 to -0.200	0.012
M.ICU	Weighted monthly HH compliance	− 0.480	− 0.826 to 0.130	0.114
	HH variability (CV)	0.370	− 0.259 to 0.778	0.237
	Critical HH day proportion (< 40%)	0.475	− 0.136 to 0.824	0.119
	Lower-tail performance (P25)	− 0.465	− 0.820 to 0.149	0.128
M.ICU II	Weighted monthly HH compliance	− 0.465	− 0.820 to 0.149	0.128
	HH variability (CV)	0.049	− 0.540 to 0.606	0.879
	Critical HH day proportion (< 40%)	0.105	− 0.499 to 0.640	0.746
	Lower-tail performance (P25)	− 0.156	− 0.670 to 0.459	0.629

Each ICU-specific analysis was based on 12 monthly observations and should therefore be interpreted cautiously. HAI incidence was expressed as HAI episodes per 100 patient-days

### Comparative interpretation of analytical approaches

Taken together, the results indicate that monthly average HH compliance remains a useful surveillance metric, as reflected by its strong pooled inverse association with HAI incidence. At the same time, complementary metrics reflecting day-to-day instability, critical low-compliance days, and lower-tail performance provided additional contextual information not captured by monthly averages alone.

The strongest pooled association was observed for weighted monthly HH compliance. Critical HH day proportion and P25 also demonstrated substantial associations with HAI incidence, whereas CV showed a weaker but still relevant association, particularly in S.ICU, where fluctuating performance appeared more closely related to HAI incidence.

Overall, these findings support the view that average compliance should not be discarded or replaced, but rather interpreted alongside additional metrics that capture operational instability and episodes of distinctly poor performance. Such a multidimensional approach may provide more nuanced information for infection prevention surveillance and management decision-making than reliance on average compliance alone, while preserving average compliance as the principal summary metric.

## Discussion

The present study provides methodological insight into how hand hygiene (HH) performance may be interpreted when examined in relation to healthcare-associated infection (HAI) incidence. Using the same prospectively collected dataset, we found that weighted monthly HH compliance remained strongly associated with HAI incidence at the pooled level and showed the strongest pooled association among the predefined metrics. At the same time, additional metrics reflecting day-to-day instability, critical low-compliance days, and lower-tail performance provided contextual information for interpreting HH performance beyond the average level alone.

These findings suggest that the relationship between HH performance and infection outcomes may depend not only on the overall level of compliance, but also on how consistently acceptable performance is maintained over time. Accordingly, the present results support a multidimensional interpretation of HH surveillance data, in which average compliance remains a central summary metric but is interpreted alongside complementary indicators of performance stability and low-compliance episodes.

### Relation to previous findings and contribution to the literature

The association between HH compliance and HAI incidence has been heterogeneous across published studies. While several investigations have reported improved HH compliance following targeted interventions, the links between measured compliance rates and infection outcomes have often been inconsistent, modest, or context-dependent [2–4].

Our findings help contextualize this heterogeneity. Rather than indicating that HH is biologically unimportant, inconsistent prior associations may partly reflect differences in how HH performance is measured, summarized, and interpreted analytically. In the present study, weighted monthly HH compliance showed the strongest pooled association with HAI incidence, while complementary metrics also contributed additional operational insight.

This observation may help reconcile previous apparently conflicting findings. Average compliance may be particularly informative in settings characterized by sustained lower performance. In contrast, instability-focused or threshold-based metrics may add contextual value in settings where average compliance appears moderate or acceptable but fluctuates substantially between observed days. Importantly, these complementary metrics should not be interpreted as replacing average compliance, but rather as helping to characterize additional dimensions of HH performance.

### Interpretation of complementary metrics

A central finding of this study is that metrics capturing repeated days of critically low performance and lower-end daily performance were associated with HAI incidence in addition to average compliance.

The proportion of critical HH days (< 40% daily compliance) may indicate months when infection prevention barriers were intermittently weakened. Such episodes may be operationally relevant even when overall monthly averages remain moderate. Similarly, the P25 metric captured the lower end of daily performance values and was less sensitive to isolated extreme observations than minimum-value metrics.

The coefficient of variation (CV) showed a weaker pooled association than weighted monthly HH compliance, critical HH day proportion, or P25. However, CV was informative in S.ICU, where performance fluctuated more markedly over time. This suggests that day-to-day instability may provide useful contextual information in selected operational settings, although it should not be interpreted independently of the underlying average compliance level.

Taken together, these findings indicate that complementary metrics should not be viewed as replacements for average compliance, but as additional descriptors of system performance. Their value lies in helping infection prevention teams identify whether similar average compliance levels reflect stable performance or are accompanied by repeated low-compliance episodes or substantial day-to-day variability.

### ICU-specific heterogeneity and workforce context

Exploratory unit-specific analyses demonstrated heterogeneous patterns across the three participating ICUs. Pooling improved statistical precision, whereas ICU-specific analyses were intended to explore contextual heterogeneity. Workforce stability and ICU work environment have previously been linked to patient safety outcomes in critical care settings [17]. In the present study, associations between HH metrics and HAI incidence were strongest in S.ICU, more moderate in M.ICU, and weakest in M.ICU II, where baseline HH compliance was generally higher and between-month variability was lower.

Several explanations are plausible. In units with consistently higher compliance and narrower performance ranges, statistical associations may be more difficult to detect because of reduced variability. Conversely, in units with more fluctuating performance, instability-related metrics may help describe periods of operational vulnerability, but these signals should be interpreted alongside the underlying average compliance level. A stable but persistently low compliance level may also plausibly contribute to higher HAI incidence.

Because the three ICUs differed in both average compliance level and performance stability, the present study cannot disentangle the independent contribution of instability from that of overall compliance level. Therefore, ICU-specific findings should be interpreted as descriptive and hypothesis-generating rather than as evidence that instability-related metrics are more important than average compliance.

Although workforce variables were not formally measured, the participating ICUs differed in staffing stability during the study period. Temporary staff deployment, onboarding of newly trained personnel, overtime burden, or varying levels of staff experience may plausibly influence day-to-day HH consistency. These factors should be regarded as contextual hypotheses rather than tested causal mechanisms, but they offer a realistic operational framework for interpreting unit-level heterogeneity.

### Practical implications for infection prevention programs

From a practical perspective, the present findings suggest that infection prevention programs may benefit from complementing average HH indicators with additional surveillance metrics.

Average monthly compliance remains a useful broad summary measure and should continue to be reported. However, monitoring the proportion of critical low-compliance days, changes in lower-tail performance, and day-to-day variability may help identify vulnerable periods that are less visible in monthly averages alone.

Such signals may support earlier and more targeted responses, including focused supervision during high-risk periods, reinforcement of HH practices during demanding shifts, temporary staffing adjustments, refresher orientation for redeployed or newly onboarded staff, and unit-specific feedback to frontline teams. These interventions should be guided by local context and interpreted together with average compliance levels rather than based on complementary metrics alone.

### Methodological implications

Several methodological considerations emerge from this study. Average HH compliance should continue to be reported as a principal summary metric, but may be complemented by additional metrics that reflect variability, critical low-compliance days, and lower-tail performance.

Threshold-based indicators should be interpreted as operational surveillance tools rather than universal clinical cut-offs. Unit-level context matters; pooled institutional analyses may obscure meaningful local heterogeneity. Direct observation datasets may yield greater value when analyzed across multiple performance dimensions rather than as single monthly averages alone. These considerations may help infection prevention teams derive more actionable insights from existing HH monitoring systems without necessarily increasing observational workload.

### Strengths

This study has several strengths. HH data were prospectively collected using a standardized WHO-based observation framework. All observations were performed by a single trained observer, improving methodological consistency across units and over time. In addition, the inclusion of three ICUs operating under shared institutional policies but with differing operational characteristics provided a useful natural comparison setting.

### Limitations

Several limitations should be acknowledged. First, analyses were conducted at the ICU-month level, limiting sample size and precluding causal inference. Second, HH observations were restricted to predefined audit days and may not fully capture unobserved daily variation. Third, direct workload, staffing, and case-mix variables were not formally measured and therefore cannot explain observed associations. Fourth, ICU-specific analyses were exploratory and based on only 12 monthly observations per unit, requiring cautious interpretation. Fifth, because the participating ICUs differed in both average compliance level and stability, the independent effects of these dimensions could not be separated. Finally, findings from a single institution may not be directly generalizable to other healthcare settings.

## Conclusions

The present findings indicate that the value of hand hygiene (HH) surveillance data depends not only on data availability, but also on how HH performance is summarized and interpreted analytically. Many institutions collect substantial volumes of HH observation data yet rely predominantly on average compliance metrics alone. Our results suggest that average monthly compliance remains an informative and central surveillance metric, while additional metrics may help characterize operationally relevant patterns that are not fully visible from averages alone.

By applying multiple predefined metrics to the same prospectively collected HH dataset, we found that weighted monthly HH compliance was strongly associated with healthcare-associated infection (HAI) incidence and showed the strongest pooled association among the metrics examined. Measures reflecting critical low-compliance days, lower-tail performance, and day-to-day instability also provided additional contextual insight. These findings suggest that inconsistent HH–HAI associations reported in previous studies may partly reflect differences in analytical approach and local context, rather than a lack of relevance of HH performance itself.

Importantly, the analytical approaches presented in this study are readily transferable. They do not require additional observation days, expanded surveillance infrastructure, or complex modeling techniques. Instead, they offer a pragmatic framework through which existing HH datasets may yield more actionable information.

For infection prevention teams and healthcare leaders, this approach may support a broader interpretation of HH surveillance data while preserving average compliance as the principal summary metric. Incorporating complementary analyses into routine surveillance may help identify periods of increased operational vulnerability and enable more timely, targeted interventions. However, these indicators should be interpreted alongside average compliance levels and local unit context rather than as standalone measures.

In summary, this study suggests that HH surveillance may be most informative when average compliance measures are supplemented by additional indicators that capture critical low-compliance days, lower-tail performance, and instability. Reconsidering how existing HH data are analyzed, rather than simply increasing data volume, may enhance infection prevention programs’ ability to translate routine observations into meaningful, context-sensitive action.

## Data Availability

The datasets generated and/or analysed during the current study are available from the corresponding author on reasonable request.
